# *Agrobacterium*-mediated transformation of *Camelina sativa* for production of transgenic plants

**DOI:** 10.14440/jbm.2018.208

**Published:** 2018-01-15

**Authors:** Viji Sitther, Behnam Tabatabai, Oluwatomisin Enitan, Sadanand Dhekney

**Affiliations:** 1 Department of Biology, Morgan State University, Baltimore, MD 21251, USA; 2 Department of Plant Sciences, Sheridan Research and Extension Center, University of Wyoming, Sheridan, WY 82801, USA

**Keywords:** direct shoot regeneration, growth regulators, plant tissue culture, transgenes

## Abstract

*Camelina sativa* (*C*. *sativa*), an oilseed species rich in poly-unsaturated fatty acids, has gained great importance as an industrial oil platform crop in recent years. Despite the potential benefits of *C. sativa* for bioenergy applications, limited research has been conducted to improve its agronomic qualities. Hence, a simple and efficient technique for production of transgenic *C. sativa* plants is warranted. In the present study, shoot apical meristems of two *C. sativa* cultivars (Pl650159 and Pl650161) were transformed with *Agrobacterium* strain ‘EHA 105’ harboring the enhanced green fluorescent protein (EGFP) and neomycin phosphotransferase II (nptII) genes. After two days of co-cultivation in the dark, explants were transferred to selection medium. Transgenic shoots were identified on the basis of green fluorescence and kanamycin resistance. Shoots were then rooted and transferred to potting mix soil for acclimatization. This protocol describes an efficient method to generate transgenic *C. sativa* plants in as little as 4 weeks.

## BACKGROUND

About 85% of our energy needs are met by petroleum-based fossil fuels such as oil, coal and natural gas. Continuous use of these nonrenewable fuels is unsustainable due to depleting supplies, rising prices, and the contribution of these fuels to atmospheric pollution [1]. Such environmental and economic concerns have driven interest in renewable bioenergy, which could serve as a substitute for fossil fuels and alleviate greenhouse gas emissions. Of the various sources of biofuel, oilseeds have recently emerged as promising sources for efficient biodiesel production. One of these crops, *Camelina sativa* (L.) (*C. sativa*) Crantz, is a member of the Brassicaceae family whose unique characteristics have fueled its rise as an important biofuel feedstock [2]. Seeds produce an oil rich in poly-unsaturated fatty acids [3,4] thereby making it a valuable renewable energy crop for the emerging biofuel industry [5,6].

**Figure 1.**
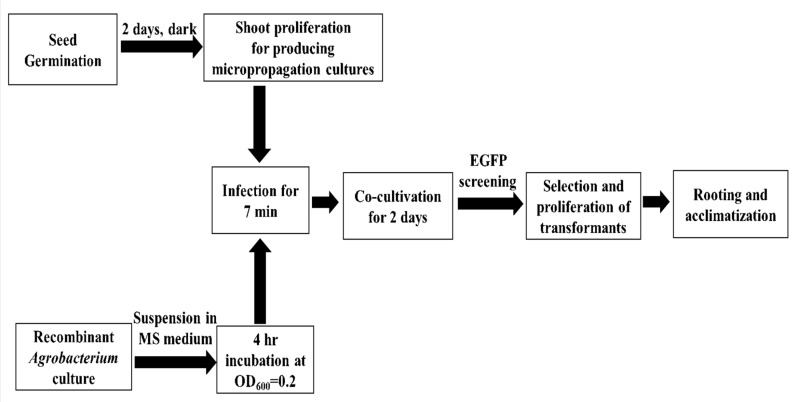
Workflow diagram of *Agrobacterium*-mediated transformation. *Camelina sativa* shoot meristems proliferated and infected with *Agrobacterium* containing binary vector. Enhanced green fluorescent protein/neomycin phosphotransferase II reporter-marker fusion system was used for selection of transformed shoots.

While *C. sativa* accessions have been investigated for their agronomic traits and oil profiles, very little research has been conducted to improve value-added qualities due to limited breeding studies [7]. Genetic transformation enables the stable incorporation of these traits resulting in varietal improvement and requires efficient *in vitro* propagation to generate a large number of transgenic plants. In addition to enhancing single traits, this process serves as an important tool for studying gene function and expression of characteristics such as disease resistance and stress tolerance. Somatic hybridization studies [7,8], plant regeneration from leaf explants [9], and floral dip transformation [10,11] have been reported; however, plant regeneration and *Agrobacterium*-mediated genetic transformation from apical and axillary meristems has not been optimized. In addition, use of shoot meristems would be more advantageous than the floral dip method, which is plagued by very low transformation efficiency [12]. In this study, we describe a transformation system for *C. sativa* using shoot apical meristem explants from rapidly growing micropropagation cultures. Transgenic plants were selected based on expression of enhanced green fluorescent protein (EGFP) and kanamycin resistance (**Fig. 1**).

## MATERIALS

### Reagents

- Bleach (Clorox^®^)
- Ethanol (Thermo Fisher Scientific, cat # S25309B)
- Tween 20 (Thermo Fisher Scientific, cat # BP337-100)
- Murashige and Skoog (1962) salts and vitamins (PhytoTechnology Laboratories, cat # M519)
- Sucrose (PhytoTechnology Laboratories, cat # S391)
- BAP (PhytoTechnology Laboratories, cat # B800)
- NAA (PhytoTechnology Laboratories, cat # N600)
- TC agar (PhytoTechnology Laboratories, cat # A111)
- Sodium hydroxide (Thermo Fisher Scientific, cat # S318-3)
- YEP (PhytoTechnology Laboratories, cat # Y8575)
- LB (PhytoTechnology Laboratories, cat # L475)
- Bactoagar (Thermo Fisher Scientific, cat # DF0140-01-0)
- Mannitol (Thermo Fisher Scientific, cat # M120-500)
- L-Glutamate (Thermo Fisher Scientific, cat # ICN19467790)
- Tryptone (Thermo Fisher Scientific, cat # BP9726-500)
- Yeast extract (Thermo Fisher Scientific, cat # AAH2676922)
- NaCl (Thermo Fisher Scientific, cat # S271-500)
- KH_2_PO_4_ (Thermo Fisher Scientific, cat # P286-1)
- MgSO_4_.7H_2_O (Thermo Fisher Scientific, cat # AC423905000)
- Fe-EDTA (Thermo Fisher Scientific, cat # AC304680051)
- Pro Mix BX potting mix (Premier Horticulture, Inc., cat # 10281RG)
- DNeasy Plant Mini kit (QIAGEN, cat # 69104)
- Primers (Integrated DNA Technologies)
- Agarose (Thermo Fisher Scientific, cat # BP160-100)
- Gel Red nucleic acid stain (Phenix Research, cat # RGB4103)
- TAE (Thermo Fisher Scientific, cat # BP13321)

### Equipment

- Biosafety cabinet class II type B2 (Nuaire, model cat # Nu-430-600) 100 × 15 mm plastic
- Petri dishes (Genesee Scientific, cat # 32-107)
- 50 ml centrifuge tube (Thermo Fisher Scientific, cat # 14-432-22)
- 125 ml conical flasks (Pyrex, cat # 4980)
- GA7 Magenta vessel (Thermo Fisher Scientific, cat # NC9357464)
- Scalpels (PhytoTechnology Laboratories, cat # S963)
- Scalpel Blade (PhytoTechnology Laboratories, cat # S970)
- Forceps (PhytoTechnology Laboratories, cat # F639)
- Sterile Whatman 3MM filter paper (Thermo Fisher Scientific, cat # 09-820A)
- Incubator shaker (VWR, model #1575)
- Centrifuge (Beckman Coulter Inc., model #J-251)
- Glass bead sterilizer (Sigma-Aldrich, model Steri-250)
- Micropipettors (Eppendorf)
- Micropipette tips (Genesee Scientific, cat # 24-120RL, 24-150RL, 24-165RS)
- 1.7 ml centrifuge tubes (Genesee Scientific, cat # 24-282)
- Conviron Growth chamber (Controlled Environments Inc., model # CMP6010)
- Zeiss Stemi SV11 microscope (Carl Zeiss AG., cat # TLB 3.1) with an X-Cite 120 fluorescence illumination system (X-Cite, model # XI-120)
- C1000 Touch Thermal PCR (Bio-Rad, model # 1851148)

### Recipes

**NOTE:** Media recipes are calculated per liter final volume. Autoclave media at 121°C and 15 psi pressure for 20 min. pH is adjusted using 1N NaOH prior to autoclaving.

- Camelina regeneration (CR) medium, pH 5.8: Murashige and Skoog (1962) salts and vitamins, 30 g/L sucrose, 6.6 μM BAP, 2.6 μM NAA, 7.0 g/L TC Agar.
- Liquid co-cultivation medium (Liquid CR), pH 5.8: Murashige and Skoog (1962) salts and vitamins, 30 g/L sucrose, 6.6 μM BAP, 2.6 μM NAA.
- Selection medium (Sel), pH 5.8: Murashige and Skoog (1962) salts and vitamins, 30 g/L sucrose, 6.6 μM BAP, 2.6 μM NAA, 7.0 g/L TC Agar, 50 mg/L cefotaxime, and 40 mg/L kanamycin.
- Solid *Agrobacterium* culture medium: yeast extract peptone (YEP) medium (10 g/L peptone, 10 g/L yeast extract, 5 g/L NaCl, pH 7.0) supplemented with 15 g/L Bactoagar.
- Liquid MG/L medium, pH 7.0: 5.0 g/L mannitol, 1.0 g/L L-Glutamate, 5.0 g/L tryptone, 2.5 g/L yeast extract, 5.0 g/L NaCl, 150.0 mg/L KH_2_PO_4_, 100.0 mg/L MgSO_4_.7H_2_O, 2.5 mg/L Fe-EDTA.

**NOTE:** MG/L medium is used for culture of *Agrobacterium* due to its ability to yield better cell growth than other bacterial media.

- Liquid MS medium, pH 5.8: Murashige and Skoog (1962) salts and vitamins.
- Camelina rooting medium: Murashige and Skoog (1962) salts and vitamins, 30 g/L sucrose, 2.1 μM NAA, 7.0 g/L TC Agar.

**Hint:** To simplify media preparation, stock solutions of 1 mg/L can be prepared separately for BAP and NAA. These chemicals can be dissolved in 1 N NaOH and diluted to final volume with distilled water.

### Plant material

- *Camelina sativa* cvs. Pl650159 and Pl650161

**NOTE:** The cultivars used in the study exhibited a high capacity for shoot regeneration.

### Antibiotic stock solutions

- Rifampicin: 20 mg of antibiotic dissolved in 500 μl of methanol.

**CAUTION:** Rifampicin is light sensitive and should be stored and used away from light.

- Kanamycin sulfate: 100 mg of antibiotic dissolved in 1 ml of water and sterilized using a 0.2 μm filter membrane.
- Cefotaxime: 100 mg of each antibiotic dissolved in 1 ml of water and filter sterilized.

**NOTE:** Antibiotic stock solutions are filter-sterilized and stored at –20°C. Antibiotics are added after autoclaving and cooling the culture medium to 55°C.

### *Agrobacterium* culture

- Binary vector containing an egfp/nptII fusion gene under the control of a cauliflower mosaic virus 35S (CaMV 35S) promoter.
- *Agrobacterium* culture stock containing the binary vector (stored in glycerol at –80°C).

## PROCEDURE

1. Initiation of micropropagation cultures from seeds
  **1.1.**Immerse seeds in 70% ethanol for 1 min.
  **1.2.**Transfer seeds to 25% commercial bleach solution containing one drop 100% Tween 20 (added using 1 ml micropipette) and surface-sterilize for 15 min with constant agitation.
  **1.3.**Rinse twice for 5 min with sterile distilled water.
  **1.4.**Blot dry seeds on filter paper and transfer to CR medium.
  **1.5.**Cover Petri dishes in aluminum foil and incubate in darkness at 25°C for 2 d.
  **1.6.**Transfer Petri dishes containing the germinated seedlings to cool white fluorescent light (75 mm m^-2^ s^-1^ and 16 h photoperiod) at 25°C for 5 d.
  **1.7.**Excise meristems from germinated seedlings and transfer to fresh CR plates under conditions described in the previous step 1.6.
    **CRITICAL STEP:** Avoid any contact of the scalpel blade with the shoot apical meristem. This could result in significant decrease or lack of proliferation in the subsequent steps.
  **1.8.**Proliferate cultures by subculture to fresh CR medium at two week intervals.
    **NOTE:** It is critical to transfer shoot cultures to fresh CR or selection (Sel) medium at 1–2 week intervals. Failure to do so will lead to a decrease in regeneration potential of explants and subsequent reduction in transformation efficiency and plant regeneration.
    **NOTE:** Use fresh shoot tips and meristems for *Agrobacterium*-mediated transformation.
2. Initiation of *Agrobacterium* culture
  **2.1.**Thaw *Agrobacterium* culture containing the binary plasmid at room temperature.
  **2.2.**Spread approximately 20 μl of bacterial culture on a Petri dish containing solid YEP medium with 20 mg/L rifampicin and 100 mg/L kanamycin.
  **2.3.**Incubate dishes in the dark at 26°C for 3 d.
  **2.4.**Isolate a single colony growing on YEP medium and transfer it to a 125 ml conical flask containing 30 ml MG/L medium with 20 mg/L rifampicin and 100 mg/L kanamycin.
  **2.5.**Incubate on a rotary shaker at 180 rpm at 26°C for 16–20 h. The bacterial culture should appear cloudy at the end of the culture period.
  **2.6.**Transfer the culture to a 50 ml centrifuge tube and spin at 6000 rpm for 8 min at room temperature. Discard the supernatant and resuspend the pellet in 30 ml liquid MS medium. Adjust optical density at 600 nm (OD_600_) value to 0.2 using liquid MS medium.
    **CRITICAL STEP:** Low OD_600_ can decrease transformation efficiency, while high OD_600_ could lead to excess growth of *Agrobacterium* and decrease virulence.
  **2.7.**Transfer the contents of the tube to a 125 ml conical flask and incubate for additional 4 h in a rotary shaker under the same conditions as above. Use this culture for co-cultivation.
3. Transformation of *C. sativa* explants
  **3.1.**Transfer shoots meristems to sterile Petri dishes.
    **NOTE:** *C. sativa* shoots proliferate by micropropagation with new shoots emerging from the primary shoot. Thus, shoot tips containing apical meristems are the best target tissues for *Agrobacterium*-mediated transformation.
  **3.2.**Add 5.0 ml *Agrobacterium* culture to explants and mix thoroughly by swirling. Incubate for 7 min. Blot explants dry on filter paper to remove the excess bacteria.
    **NOTE:** Blot-drying explants on filter paper is beneficial as it reduces excessive growth of bacterial cells resulting in a dramatic decrease in cell necrosis and higher transformation efficiency.
  **3.3.**Transfer blot-dried explants to solid CR medium.
  **3.4.**Seal the Petri dish with Parafilm^®^ and co-cultivate in darkness at 26°C for 2 d.
  **3.5.**After 2 d observe explants for transient GFP expression using microscope equipped with fluorescence illumination system.
  **3.6.**Transfer co-cultivated explants to petri dishes containing solid Sel medium.
  **3.7.**Transgenic cultures can be identified on the basis of GFP fluorescence and kanamycin resistance and can be used to separate transgenic lines from non-transformed cultures. Designate each line as an independent event and transfer to Sel medium.
  **3.8.**Transfer shoots to Magenta GA7 vessels containing 30 ml Camelina rooting medium. Place vessels under conditions previously mentioned in step 1.6.
  **3.9.**Transfer plants to 7 cm plastic pots containing Pro Mix BX potting mix and acclimatize in a growth room for two weeks before transfer to a greenhouse.
4. Molecular analyses of transgenic plants
  **4.1.**Total genomic DNA can be isolated using the QIAGEN DNeasy Plant Mini Kit.
  **4.2.**The presence of the transgenes can be confirmed by PCR using gene specific primers for GFP (5’- ATGGTGAGCAAGGGCGAGG AGCTGT-3’ and 5’- CTTGTACAGCTCGTCCATGCCGAGA-3’) and NPT II (5’- CGGCCG CTTGGGTGGAGAGG CTATT-3’ and 5’- TCAGAAGAACTC GTCAAGAAGGCGA -3’). PCR reactions are carried out under the following conditions: 1 cycle at 95°C for 4 min, 40 cycles at 94°C for 1 min, 58°C for 1 min, 72°C for 1 min and a final cycle at 72°C for 4 min.
  **4.3.**PCR products can be visualized by agarose gel electrophoresis.

### ANTICIPATED RESULTS

Improvement of *C. sativa* germplasm for breeding programs requires the development and establishment of *in vitro* regeneration and transformation methods. Although the possibility of apical shoot meristems for plant regeneration and genetic transformation has long been suggested in other crops [13], there is no previous report on an egfp/nptII reporter-marker fusion transformation in *C. sativa*.

**Figure 2.**
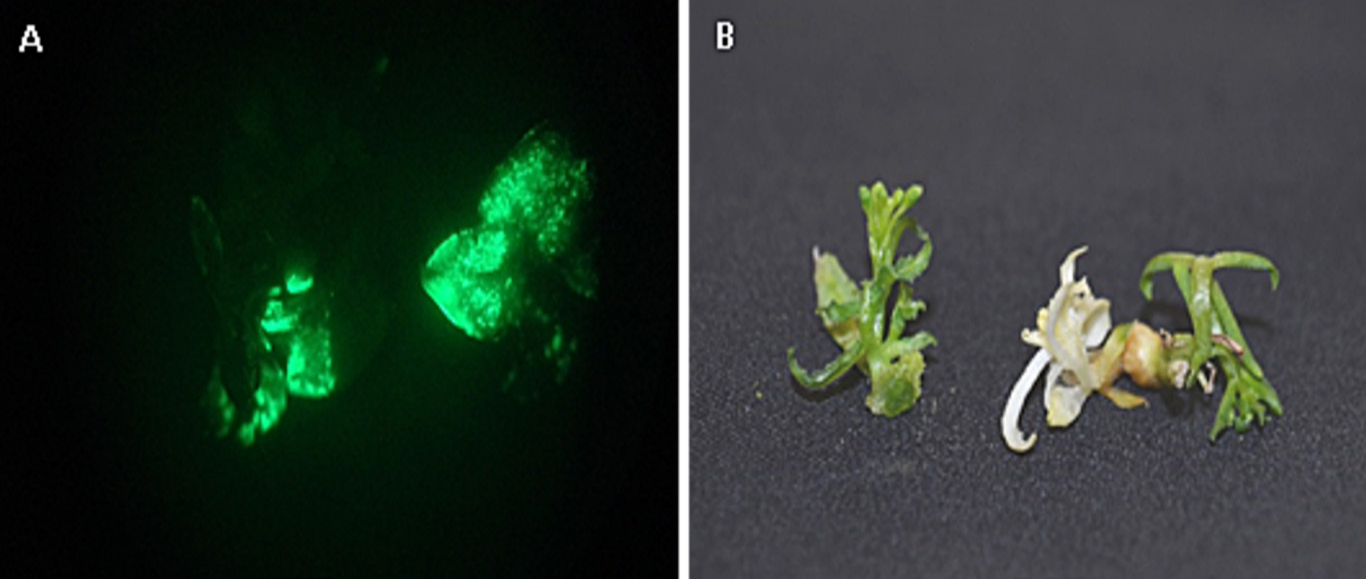
Selection of transformed *Camelina sativa* shoot meristems. **A.** Green fluorescence protein expression in *C. sativa* apical meristems. **B.** Non-transgenic (left) in *C. sativa* regeneration media (no kanamycin) and transgenic (right) shoots after seven days in selection media (40 mg/L kanamycin).

The procedure described herewith offers an optimized method of *Agrobacterium*-mediated transformation of apical shoot cultures as monitored by EGFP expression. Shoot tips that expressed EGFP produced a bright green fluorescence when observed under a microscope equipped with epi-fluorescence illumination (**Fig. 2A**). Additionally, transgenic cells carrying these marker genes selectively grew on culture medium containing kanamycin while inhibiting the growth of non-transformed cells (**Fig. 2B**). Selectable marker genes such as npt II and hygromycin phosphotransferase are routinely used along with reporter genes in genetic transformation [14].

**Figure 3.**
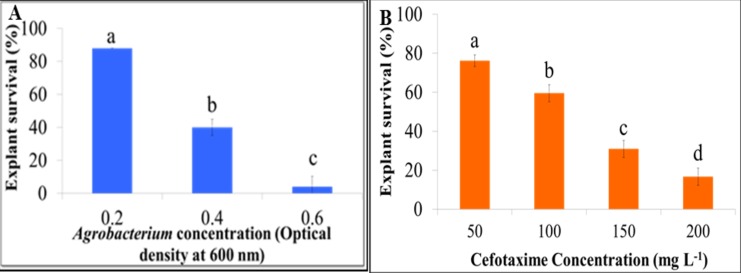
Optimization of *Camelina sativa Agrobacterium*-mediated transformation protocol. **A.** Effect of *Agrobacterium* concentration (optical density at 600 nm) on survival of *C. sativa* shoot explants. **B.** Effect of cefotaxime concentration on *C. sativa* explant survival. Different letters above bars indicate significance among treatment means (*P* < 0.05) based on ANOVA and Tukey’s HSD post-hoc test.

**Figure 4.**
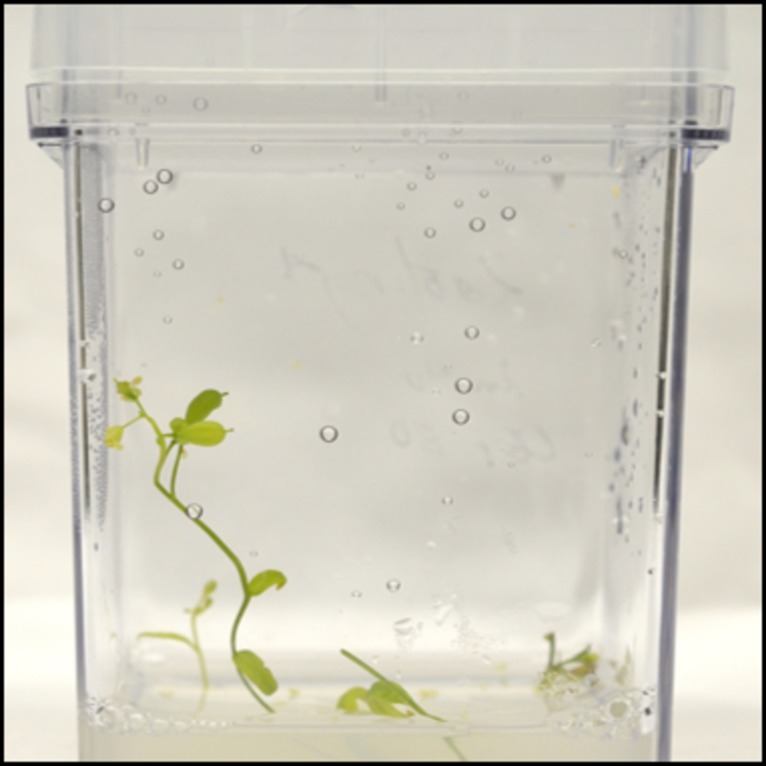
Transgenic *Camelina sativa* shoots on rooting medium containing kanamycin for root development.

Transformation with an initial *Agrobacterium* optical density (OD_600_) of 0.6 was excessive, while a reduction to 0.2 resulted in increased explant survival following cocultivation (**Fig. 3A**). In addition, reduction of co-cultivation interval from 3 to 2 d and a cefotaxime concentration to 200 to 50 mg/L (**Fig. 3B**) enhanced the survival of explants in both cultivars. A co-cultivation period of 3 d resulted in the death of all explants due to excess *Agrobacterium* growth. This report details a novel and efficient protocol for *Agrobacterium*-mediated transformation of *C. sativa* shoot meristems to generate transgenic plants in as little as 28 d (**Fig. 4**). Optimization of parameters for genetic transformation is essential for explant proliferation and improvement of value-added traits in plants [15].

## TROUBLESHOOTING

Potential problems and troubleshooting suggestions are listed in **Table 1**.

## Figures and Tables

**Table 1.** Troubleshooting.

Step	Problem	Cause	Suggestions
1.1–1.4	Seed not germinating	Overexposure to ethanol or bleach Seed dried out from blot drying Seed damaged by handling	Make sure not to exceed maximum time during surface sterilization Be gentle while blot drying (step 1.4) or eliminate step completely
1.6, 1.7	Meristem cultures not proliferating	Improper media preparation Meristem damage Explants too small	Carefully screen every step of the media preparation process, particularly pH and plant growth regulators Avoid cutting near meristem Subculture with larger explants
2.5	Liquid culture not growing or contaminated	Source bacterial culture is old Media is contaminated	Streak a fresh plate every 10 d to ensure source plate is active Aliquot liquid media in smaller bottles to minimize contamination
3.5	No GFP expression observed	Transformation did not work	Use smaller explants for transformation Check previous steps carefully, especially step 2.6 Lightly wound leaves with dull side of scalpel blade
